# Coronavirus disease 2019 vaccination uptake and hesitancy among Polish patients with inborn errors of immunity, autoinflammatory syndromes, and rheumatic diseases: A multicenter survey

**DOI:** 10.3389/fimmu.2022.1010899

**Published:** 2022-10-06

**Authors:** Ewa Więsik-Szewczyk, Marcin Ziętkiewicz, Agata Będzichowska, Katarzyna Napiórkowska-Baran, Aleksandra Matyja-Bednarczyk, Anna Felis-Giemza, Karina Jahnz-Różyk

**Affiliations:** ^1^ Department of Internal Medicine, Pneumonology, Allergology and Clinical Immunology, Central Clinical Hospital of the Ministry of National Defense, Military Institute of Medicine, Warsaw, Poland; ^2^ Department of Internal Medicine, Connective Tissue Diseases and Geriatrics, Medical University of Gdansk, Gdansk, Poland; ^3^ Department of Pediatrics, Pediatric Nephrology and Allergology, Central Clinical Hospital of the Ministry of National Defense, Military Institute of Medicine, Warsaw, Poland; ^4^ Department of Allergology, Clinical Immunology and Internal Diseases, Ludwik Rydygier Collegium Medicum in Bydgoszcz Nicolaus Copernicus University in Torun, Bydgoszcz, Poland; ^5^ 2nd Department of Internal Medicine, Jagiellonian University Medical College, Krakow, Poland; ^6^ Biologic Therapy Center, National Institute of Geriatrics, Rheumatology and Rehabilitation, Warsaw, Poland

**Keywords:** inborn errors of immunity (IEI), autoinflammatory syndromes, autoimmune inflammatory diseases, COVID-19 vaccination, biologic treatment

## Abstract

Data regarding the willingness of patients affected by inborn errors of immunity to accept vaccination against severe acute respiratory syndrome coronavirus 2 (SARS-CoV-2) infection are limited. Therefore, this study assessed SARS-CoV-2 vaccination coverage and hesitancy in immunodeficient patients by surveying adults with primary immune deficiencies and autoinflammatory and rheumatic diseases on biologic therapy. The study was conducted from September 20, 2021, to January 22, 2022, when the primary coronavirus disease 2019 (COVID-19) vaccinations were available to all adults in Poland. We included 207 participants consecutively recruited from five referral centers (57% female; median age: 42.6 [range: 18–76, standard deviation ± 14.70] years). Overall, 55% (n = 114), 17% (n = 36), and 28% (n = 57) of the patients had primary immune deficiencies, autoinflammatory diseases, and rheumatic diseases, respectively. Among the entire cohort, 168 patients (81%) were vaccinated, and 82% were willing to receive a booster dose. Patients with autoinflammatory diseases had the highest vaccination rate (94.4%). A strong conviction that it was the correct decision (72%), fear of getting COVID-19 (38%), and expert opinions (34%) influenced the decision to vaccinate. Among the unvaccinated patients, 33.3% had primary or vocational education (p <0.001). Furthermore, only 33% believed they were at risk of a severe course of COVID-19 (p = 0.014), and 10% believed in vaccine efficacy (p <0.001). They also doubted the safety of the vaccine (p <0.001) and feared a post-vaccination flare of their disease (p <0.001). Half of the unvaccinated respondents declared that they would consider changing their decision. Vaccination coverage in immunodeficient patients was higher than in the general Polish population. However, the hesitant patients doubted the vaccine’s safety, feared a post-vaccination disease flare, and had primary or vocational education. Therefore, vaccination promotion activities should stress personal safety and the low risk of disease flares due to vaccination. Furthermore, all evidence must be communicated in patient-friendly terms.

## Introduction

Mass prophylactic vaccinations are currently the only effective strategy against a severe course of coronavirus disease 2019 (COVID-19). Regulatory COVID-19 vaccine trials generally excluded immunocompromised patients, enrolling only a few with cancer or autoimmune diseases (1). Therefore, this group of patients may have a conflicted attitude towards vaccination due to limited population-specific vaccine safety data. From the pandemic’s outset, adult immunocompromised patients, including those with inborn errors of immunity (IEI), were identified as a vulnerable population with a high risk of developing severe COVID-19. Thus, they have been prioritized in the Polish vaccination program. Furthermore, in Poland, a negative attitude towards vaccinations has limited the possibility of achieving herd immunity (2–4). Between January and April 2021, the percentage of adult Poles who declared a negative attitude towards the COVID-19 vaccine and a lack of willingness to vaccinate against COVID-19 remained at a stable level of 31%, despite the implementation of educational programs, media campaigns, and vaccination promotions by public authorities and medical professionals (3). Data from October 2021 showed that only 53% of Poles were vaccinated with at least one dose, putting Poland in 23^rd^ place for vaccination rates among countries in the European Union (5).

Studies have reported vaccine hesitancy in 13.4%, 19.4%, and 17.8% of patients with cancer, autoimmune diseases, and chronic lung diseases, respectively (6), despite expert opinions that the benefits of being vaccinated outweigh the limitations of available evidence for a specific high-risk group (7–10). Moreover, vaccine resistance appears to correlate with geographic location, country-specific regulations of vaccines availability, the proposed vaccination schedule, study methodologies, the study period in relation to the pandemic wave, and evolving vaccine safety and efficacy data (1, 11–16). For example, 54% of patients with autoimmune inflammatory rheumatic diseases (AIIRDs) initially accepted the idea of COVID-19 vaccinations (17). However, with time, self-reported vaccine acceptance among patients with AIIRDs increased from 62% in December 2020 to 94% in August 2021 (18). Finally, in 2022, 80% of patients with AIIRDs self-confirmed that they were vaccinated (19). However, the data based on online research reports may be overstated or biased due to an underrepresentation of persons with low income, lower education, or limited access to the internet, such as in rural areas. Moreover, vaccinated individuals have been more willing to complete vaccination surveys than unvaccinated individuals (19).

Emerging reports on vaccine safety and efficacy in the AIIRD and IEI populations should be sufficient to promote vaccination (20–23). However, nocebo-prone attitudes remain (24), and the daily vaccination pace has plateaued and even declined (25). Thus, understanding why those in high-risk groups continue to forego COVID-19 vaccinations is even more important.

In contrast to other chronic diseases and at-risk populations, vaccine hesitancy and willingness data is limited among those affected by IEI. For example, we only identified one Canadian study that used the SurveyMonkey Internet-based questionnaire platform conducted from April to May 2021 with a 40% response rate (26). Furthermore, the International Patient Organization for Primary Immunodeficiencies conducted a survey and presented the results during a November 2021 webinar organized by the European Society for Immunodeficiencies (ESID), but Polish patients were not represented (27).

Therefore, we addressed vaccine hesitancy among adult patients with IEI and autoinflammatory diseases (AIDs) in Poland, including those with AIIRDs treated with biologics, to clarify the attitude towards COVID-19 vaccination and explore differences between those with primary and secondary immunodeficiencies.

## Materials and methods

### Study population

We conducted an on-site survey on vaccination attitudes against COVID-19 among adult patients diagnosed with IEI and AIDs and patients with AIIRDs receiving targeted biologic therapy. All the included patients had scheduled in-person follow-up visits at reference centers. The study was performed from September 20, 2021, to January 22, 2022, primarily during the fourth COVID-19 wave of the pandemic. During this period, the number of reported daily deaths from COVID-19 in Poland was more than 500 (28). At the same time, the initial COVID-19 vaccination course was available to all adults in Poland, and in October 2021, the Polish Ministry of Health prioritized the third booster dose for select risk groups, including adult patients with significant immunodeficiencies independently of etiology (29).

### Data collection

The survey included the following data: age, sex, clinical diagnosis, treatment, comorbidities (yes or no), COVID-19-related experiences, COVID-19 vaccination history, seasonal influenza vaccination history; COVID-19 vaccine booster intention (yes or no), and fears and expectations about the COVID-19 vaccination (**Table 1**). Also, the Brief Illness Perception Questionnaire was used to assess illness perception (30, 31). The supplementary materials contain the full-length questionnaire (**Table S1**).

**Table 1:** Representative questions from the Vaccine Hesitancy Questionnaire.

Fear of SARS-Cov-2 infection was searched using the question	
Do you think that you are at risk of severe COVID 19 course? Yes No I do not know. To address patients attitudes, opinions and hesitancy following questions were used: What influenced your decision to vaccinate (multiple options can be chosen)? My own opinion that it is correct decision Fear of getting COVID-19 Expert opinion Opinion of relatives and friends Other In your opinion vaccination against COVID-19 is safe? Yes Yes, but only for healthy people I do not know No. In your opinion vaccination against COVID-19 is effective in persons with your disease? Yes I don’t know No Are you afraid that vaccination against COVID-19 may flare/worsen your diseases? Yes No I do not know To address cocoon strategy we asked patients if in their opinion Should COVID-19 vaccination be mandatory? (you can choose multiple answers)? No. Yes, for everyone Yes, but only for selected professional groups Yes, for people at risk of severe disease course

### Statistical analyses

The normality of the observed values was tested using the Shapiro-Wilk test. Continuous variables were analyzed using Mann-Whitney-U, Student’s t, or Kruskal-Wallis tests. Categorical variables were analyzed using the Chi-square or Fisher’s exact test. For all analyses, differences were considered statistically significant when the p-value was <0.05. Statistical analyses were performed using Statistica, version 13 (TIBCO Software Inc.).

### Ethics statement

The Military Institute of Medicine Ethics Committee, Warsaw, Poland, approved this study (No. 31/WIM/2021). All patients provided written consent to collect and analyze their demographic and medical data.

## Results

### Population characterization

We invited 213 patients to participate; 6 patients (2%) refused (1 patient was diagnosed with primary immune deficiency, and 5 were diagnosed with chronic arthritis). Finally, we included 207 participants. The median age was 42.6 years (range: 18–76, mean 42 ± 14.70 years), and 118 were women (57%). The patients were recruited from five referral centers (four IEI centers and one rheumatology center with biologic treatments).

We included 114 patients (114/207, 55%) with IEI diagnosed based on the ESID guidelines, including common variable immunodeficiency (n = 53), agammaglobulinemia (n = 10), subclass deficiencies (n = 10), isolated immunoglobin A deficiency (n = 1), specific antibody deficiency (n = 1), unclassified hypogammaglobulinemia (n = 3), Wiskott-Aldrich syndrome (n = 1), autoimmune lymphoproliferative syndrome (n = 2), Nijmegen breakage syndrome (n = 1), Bloom syndrome (n = 2), and DiGeorge syndrome (n = 1). The most common treatment was human immunoglobulin replacement therapy (108/114 patients, 95%). In total, 98 (91%) and 10 (9%) of 108 patients underwent at-home subcutaneous immunoglobulin therapy and in-patient intravenous immunoglobulin, respectively. Only two brothers with X-linked agammaglobulinemia were familial cases among those with IEIs.

The AID group included 36 patients (36/207, 17%). The diagnoses included NLR family pyrin domain containing 3 (NLRP3)-related diseases (n = 12), tumor necrosis factor receptor-associated periodic syndrome (i.e., TRAPS, n = 9), familial Mediterranean fever (n = 3), mevalonate kinase deficiency (n = 2), undifferentiated systemic AIDs (n = 5), and Schnitzler syndrome (n = 5). Overall, 23 (61%) and 13 (39%) of 36 cases were sporadic and familial, respectively. The familial cases included five families: 1) a trio of two sisters and one of their daughters, 2) a trio of a mother and two daughters, 3) a trio of a mother with one son and one daughter, 4) a father and son pair, and 5) a mother and son pair. At the time of the survey, 32 of 36 patients with AIDs (89%) took anakinra, a short-lasting interleukin-1 inhibitor.

Finally, the AIIRD group included 57 patients (57/207, 28%), including confirmed rheumatoid arthritis (n = 18), ankylosis spondylitis (n = 30), psoriatic arthritis (n = 9), and juvenile idiopathic arthritis (n = 2). All were treated with biologic agents. The most common treatments were tumor necrosis factor-alpha inhibitors (n = 48, 84%) followed by interleukin-6 (n = 6, 10.5%) and interleukin-17 (n = 3, 5%) inhibitors.

Overall (n = 207), 122 patients (60%) had other comorbidities or chronic conditions, but these did not differ among the groups (**Table 2**). Moreover, 53 patients (25%) lived in rural areas; 29 patients (14%) had primary and vocational education, 77 (37%) had secondary education, and 101 (49%) had higher-level education. Furthermore, 50% of the patients had been vaccinated against influenza, including 23.7% who were vaccinated annually; patients with AIIRDs had the lowest influenza vaccination rate (**Table 2**). Age, sex, education, living area, and COVID-19 history did not differ among the groups (**Table 2**), but their disease perception did; patients with AIIRDs anticipated the worst effects of the disease on their lives. **Table 3** presents the Brief Illness Perception Questionnaire results and comparisons.

**Table 2:** Comparisons of select features among the study groups.

	Total (N = 207)	Analyzed groups according to diagnosis				Vaccination status		
		AID (N = 36)	IEI (N = 114)	AIIRDs (N = 57)	p	NO (N = 39)	YES (N = 168)	P
**Age Mean ± SD**	42.6 ± 14.8	40.6 ± 14.2	41.4 ± 15.3	46.3 ± 13.6	0.064	38.6 ± 15.8	43.6 ± 14.4	0.053
**Education**								
Primary	9 (4.3 %)	2 (5.6 %)	6 (5.3 %)	1 (1.8 %)	0.061	6 (15.4 %)	3 (1.8 %)	**<0 .001**
Vocational	20 (9.7 %)	6 (16.7 %)	13 (11.4 %)	1 (1.8 %)		7 (17.9 %)	13 (7.7 %)	
Secondary	77 (37.2 %)	13 (36.1 %)	45 (39.5 %)	19 (33.3 %)		12 (30.8 %)	65 (38.7 %)	
Higher	101 (48.8 %)	15 (41.7 %)	50 (43.9 %)	36 (63.2 %)		14 (35.9 %)	87 (51.8 %)	
**Sex**								
Female	118 (57.0 %)	18 (50.0 %)	63 (55.3 %)	37 (64.9 %)	0.314	20 (51.3 %)	98 (58.3 %)	0.423
Male	89 (43.0 %)	18 (50.0 %)	51 (44.7 %)	20 (35.1 %)		19 (48.7 %)	70 (41.7 %)	
**Residence**								
City	154 (74.4 %)	25 (69.4 %)	84 (73.7 %)	45 (78.9 %)	0.573	28 (71.8 %)	126 (75.0 %)	0.679
Village	53 (25.6 %)	11 (30.6 %)	30 (26.3 %)	12 (21.1 %)		11 (28.2 %)	42 (25.0 %	
**Co-morbidities**								
Missing response	2 (1.0 %)	0 (0.0 %)	2 (1.8 %)	0 (0.0 %)	0.102	0 (0.0 %)	2 (1.2 %)	0.125
No	83 (40.1 %)	18 (50.0 %)	37 (32.5 %)	28 (49.1 %)		21 (53.8 %)	62 (36.9 %)	
Yes	122 (58.9 %)	18 (50.0 %)	75 (65.8 %)	29 (50.9 %)		18 (46.2 %)	104 (61.9 %)	
**Do you think that you are at risk of severe COVID 19 course?**								
Missing response	6 (2.9 %)	2 (5.6 %)	2 (1.8 %)	2 (3.5 %)	0.111	0 (0.0 %)	6 (3.6 %)	**0.017**
No	96 (46.4 %)	22 (61.1 %)	48 (42.1 %)	26 (45.6 %)		26 (66.7 %)	70 (41.7 %)	
Yes	105 (50.7 %)	12 (33.3 %)	64 (56.1 %)	29 (50.9 %)		13 (33.3 %)	92 (54.8 %)	
**Did you suffer from COVID-19 disease?**								
No	142 (68.6 %)	25 (69.4 %)	80 (70.2 %)	37 (64.9 %)	0.612	24 (61.5 %)	118 (70.2 %)	0.138
Yes, at home	51 (24.6 %)	9 (25.0 %)	24 (21.1 %)	18 (31.6 %)		10 (25.6 %)	41 (24.4 %)	
Yes, in hospital	11 (5.3 %)	1 (2.8 %)	8 (7.0 %)	2 (3.5 %)		3 (7.7 %)	8 (4.8 %)	
I do not know	3 (1.4 %)	1 (2.8 %)	2 (1.8 %)	0 (0.0 %)		2 (5.1 %)	1 (0.6 %)	
**Have your family members/housemates suffered from COVID-19?**								
No	121 (58.5 %)	18 (50.0 %)	69 (60.5 %)	34 (59.6 %)	0.288	19 (48.7 %)	102 (60.7 %)	0.274
Yes, at home	78 (37.7 %)	16 (44.4 %)	39 (34.2 %)	23 (40.4 %)		19 (48.7 %)	59 (35.1 %)	
Yes, in hospital	8 (3.9 %)	2 (5.6 %)	6 (5.3 %)	0 (0.0 %)		1 (2.6 %)	7 (4.2 %)	
**Have you been vaccinated against influenza?**								
Sometimes	56 (27.1 %)	12 (33.3 %)	35 (30.7 %)	9 (15.8 %)	**<0 .001**	9 (23.1 %)	47 (28.0 %)	**<0 .001**
Never	102 (49.3 %)	19 (52.8 %)	43 (37.7 %)	40 (70.2 %)		29 (74.4 %)	73 (43.5 %)	
Yes, yearly	49 (23.7 %)	5 (13.9 %)	36 (31.6 %)	8 (14.0 %)		1 (2.6 %)	48 (28.6 %)	

Bold means that they are statistically significant.

**Table 3:** Brief Illness Perception Questionnaire result comparisons among the study groups.

	Total (N = 207)	Analyzed groups according to diagnosis				Vaccination status		
		AID (N = 36)	IEI (N = 114)	AIIRDs (N = 57)	p	NO (N = 39)	YES (N = 168)	p
**How much does your illness affect your life?**	5.671 ± 2.65	5.889 ± 2.785	5.237 ± 2.594	6.404 ± 2.542	**0.029**	5.821 ± 3.025	5.637 ± 2.565	0.689
**How long do you think your illness will continue?**	9.541 ± 1.36	8.944 ± 2.216	9.746 ± 0.860	9.509 ± 1.390	0.157	9.436 ± 1.071	9.565 ± 1.421	0.075
**How much control do you feel you have over your illness?**	5.594 ± 2.79	6.167 ± 3.402	6.035 ± 2.566	4.351 ± 2.416	**<0 .001**	5.487 ± 2.470	5.619 ± 2.860	0.740
**How much do you think your treatment can help your illness?**	7.304 ± 2.18	7.889 ± 2.240	7.342 ± 2.173	6.860 ± 2.108	**0.024**	7.205 ± 2.142	7.327 ± 2.198	0.646
**How much do you experience symptoms from your illness?**	5.005 ± 2.53	4.639 ± 3.006	4.746 ± 2.551	5.754 ± 1.994	0.052	5.128 ± 2.697	4.976 ± 2.498	0.470
**How concerned are you about your illness?**	4.643 ± 2.98	5.250 ± 3.219	3.851 ± 2.810	5.842 ± 2.691	**<0 .001**	4.641 ± 2.969	4.643 ± 2.990	0.970
**How well do you feel you understand your illness?**	7.213 ± 2.43	6.833 ± 2.864	7.333 ± 2.349	7.211 ± 2.320	0.745	7.103 ± 2.521	7.238 ± 2.418	0.768
**How much does your illness affect you emotionally? (e.g. does it make you angry, scared, upset or depressed)?**	4.807 ± 2.82	4.667 ± 2.704	4.404 ± 2.793	5.702 ± 2.784	**0.020**	5.179 ± 3.292	4.720 ± 2.700	0.351

Bold means that they are statistically significant.

### COVID-19 history and vaccination against severe acute respiratory syndrome coronavirus 2 (SARS-CoV-2)

In total (n = 207), 62 patients (30%) suffered from COVID-19, including: 34 patients (29%) with IEIs, 10 (27%) with AIDs, and 20 (35%) with AIIRDs. Of these patients, 10 (17%) were hospitalized due to COVID-19 (8 with IEIs and 2 with AIIRDs). One patient with AIDs become SARS-CoV-2-positive during hospitalization due to a Schnitzler syndrome flare.

Moreover, 168 patients (81.2%) were vaccinated (IEI: n = 89 7,8.1%; AID: n = 34, 94.4%; and AIIRD: n = 45, 78.9%); the proportion of vaccinated patients did not differ among the groups (**Table 2**). The participants were vaccinated with BNT162b2 (n =126, 75.0%), mRNA-1273 (n = 16, 9.5%), AZD1222 (n = 15, 8.9%), and Ad26.COV2.S (n = 10, 6.0%). One response was missing. Side effects occurred in 59 patients (35.1%). Three severe events occurred in 3 patients, all of which had AIDs; 1 patient was hospitalized due to prolonged fever, 1 patient was observed in the emergency unit due to convulsions, and 1 had facial palsy requiring long-term rehabilitation. The other events were mild, moderate, and local-side reactions.

The decision to vaccinate was influenced by the strong conviction that receiving the vaccination was the correct decision (n = 121, 72.0%), a fear of getting COVID-19 (n = 78, 46.4%), expert opinions (n = 71, 42.3%), and the opinion of relatives (n = 15, 8.9%). The responders could provide multiple answers. Most vaccinated participants were willing to receive the booster dose (n = 132, 82%). Overall, 110 of 207 patients (53.1%) believed vaccination against COVID-19 should be obligatory.

### Unvaccinated and vaccinated patient comparisons

We compared the unvaccinated (n = 39) and vaccinated (n = 168) patients. Among the unvaccinated individuals, 13 (33.3%), 12 (30.8%), and 14 (35.9%) patients had primary or vocational education, secondary education, and higher education, respectively. Among the vaccinated individuals, 16 (9.5%), 65 (38.7%), and 87 (51.8%) patients had primary or vocational education, secondary education, and higher education, respectively. The number of individuals with primary or vocational education significantly differed between the unvaccinated and vaccinated groups (p <0.001).

Significantly fewer unvaccinated patients believed they were at risk for a severe course of COVID-19 (n = 13, 33.3% vs. n = 92, 56.7%, p = 0.014). Most unvaccinated patients did not believe that vaccine was effective given their condition (n = 35, 89.7%); this attitude significantly differed from the vaccinated individuals (n = 119, 70.8%; p <0.001).

When asked if vaccination is safe, significantly more unvaccinated individuals answered “No or I don’t know” (n = 29, 74.4%) than vaccinated individuals (n = 39, 23.2%; p <0.001). More unvaccinated than vaccinated individuals believed that the vaccine was only safe for healthy individuals (n = 8, 20.5% vs. n = 3, 1.8%; p <0.001). Furthermore, significantly more unvaccinated individuals feared a disease flare after vaccination than vaccinated individuals (n = 30, 76.9% vs. n = 13, 7.7%; p <0.001).

In the unvaccinated group, 44% of respondents did not answer the question about what drove their vaccination decision compared to 0.6% of vaccinated respondents. In addition, half of the unvaccinated patients declared they could change their minds in the future. Sex, age, residence, COVID-19 history, comorbidities, treatment regimen, and disease perception did not differ between the unvaccinated and vaccinated groups.

## Discussion

In this study, 80% of patients with IEI, AIDs, and AIIRDs were vaccinated against COVID-19 compared to 54% in the general Polish population. The difference is even more striking when considering the mean age (32, 33). We confirmed that the COVID-19 vaccine acceptance rate was similar among the IEI, AID, and AIIRD groups. Furthermore, we found that vaccine hesitancy was primarily due to doubts about the vaccine’s efficacy, safety, and flares of their underlying disease. Moreover, the decision not to vaccinate strongly correlated with primary or vocational education. However, half of the unvaccinated patients declared that they would consider changing their opinion on vaccinations.

Our results are similar to the Canadian study of IEI patients and a study that included patients with AIIRDs (15, 26). In our study, patients with AIDs had the highest vaccination rate (94%), but we could not identify a previous study targeting this patient population. Therefore, despite this group’s limited data on prophylactic vaccination, a high COVID-19 vaccination acceptance rate among patients with AIDs occurred (34, 35). Moreover, flares of NLRP3-related AIDs were documented after the pneumococcal vaccine (35). We propose that familial aggregation of monogenic AIDs and similar decisions among familial cases contributed to the high vaccination rate in this group. This result may also be attributed to the policies and experience of the patient’s referral centers, where the benefits and risks of vaccination were discussed in a personalized way and supported by the health care provider’s expertise. In our study, only 50% of all patients received an influenza vaccine, with the lowest uptake among patients with AIIRDs (30%), consistent with other studies (36). However, in Poland, seasonal influenza vaccine administration is extremely low in the general population, with only 6% and 9% of individuals undergoing vaccination in the 2020/2021 and 2021/2022 seasons, respectively (37, 38).

In this study, 30% of patients answered that the experts’ opinions were important for their decision to be vaccinated. This agrees with the study in patients with AIIRDs, where 34% of vaccinated individuals attributed their decision to advice from their physician (18), Trusting relationships with physicians can decreases vaccine hesitancy (39) by stressing the personal benefits (40). This finding emphasizes the importance of vaccination-specific counseling to improve COVID-19 vaccine coverage, which might also be relevant for other vaccines, such as influenza or pneumococcal (41). However, pharmacist-physician coaching (42) might be ineffective among patients with ultrarare and rare diseases since they generally seek professional advice from a specialist before receiving any vaccines.

Our data on vaccine hesitancy also agrees with the Canadian study results that reported the primary reason for vaccine hesitancy was uncertainty of the benefits (26). However, other studies reported safety concerns among patients with IEI or AIIRDs. Some studies evaluated the specific patient concerns related to the COVID-19 vaccine, which included unknown long-term side effects, the newness of the COVID-19 vaccine, and the perception of rushed development and introduction with potential financial links to the pharmaceutical companies (9, 18, 24, 26). Those unvaccinated also feared that the vaccine would be harmful and could cause thrombosis (24), despite well-documented contrary evidence (43). Furthermore, a concern about potential side effects is a well-documented primary argument against COVID-19 vaccination among the general Polish population (3) and health care workers, especially nurses (4).

Flares of the existing disease due to prophylactic vaccination is another strong belief and stereotype among patients with systemic autoimmune diseases. Both patients and health care professionals present concerns about interactions with immunosuppressive treatment regimens or the underlying immune-mediated inflammatory disease. Consequently, these arguments become a more prominent reason for doubt or refusal over time (24, 41).

In our study, the decision not to vaccinate strongly correlated with primary or vocational education. This result is supported by studies performed in different countries (6, 17, 24). In our opinion, this result underscores the necessity to communicate the scientific results and arguments for vaccination in patient-friendly language.

In contrast to other studies on patients with IEI, half of the unvaccinated participants in this study declared the possibility of changing their choice (26). A study performed before introducing the vaccination program in the Netherlands presented a “watch and wait” strategy for patients unsure about vaccination. According to public health records, this approach possibly reduced vaccine hesitancy (44); thus, we agree with this strategy. However, patient opinions towards vaccination should be periodically reevaluated to address the risk of nocebo-prone attitudes.

This study has some limitations. For example, we included a small number of patients. However, the included patients had rare conditions, and their diagnoses and immunodeficiency states were confirmed by health care professionals based on accepted criteria and not self-reported. Nonetheless, we used a self-prepared questionnaire and did not include questions addressing a nocebo-prone attitude, which may be important in a vaccine hesitancy analysis. We also only included patients within referral centers that strongly promote prophylactic vaccination against COVID-19 for high-risk groups, which may have increased the number of vaccinated respondents.

## Conclusions

To our knowledge, this is the first study on vaccine hesitancy in patients with IEI from central Europe, including a considerable proportion of AIDs patients. Also, our study is unique because we performed on-site surveys on consecutive patients. Thus, we obtained a very high response rate from patients with rare diseases.

The percentage of vaccinated persons in each subgroup was higher than that in the general Polish population. Unvaccinated patients doubted the efficacy and safety of the vaccine and were afraid of flares of their underlying disease after vaccination but did not fear a severe course of COVID-19; approximately one-third had only primary or vocational education. Nonetheless, despite their hesitancy, half of the unvaccinated respondents declared the possibility of changing their decision. Our findings suggest that vaccine promotion activities should stress personal safety. Furthermore, patients must be informed about the low risk of disease flares due to vaccination, and all evidence must be updated and communicated in patient-friendly language. Finally, these findings emphasize the importance of vaccination-specific counseling to improve COVID-19 vaccine coverage, which might also be relevant for influenza and pneumococcal vaccinations.

## Data availability statement

The raw data supporting the conclusions of this article will be made available by the authors, without undue reservation.

## Ethics statement

The studies involving human participants were reviewed and approved by The Military Institute of Medicine Ethics Committee, Warsaw, Poland. The patients/participants provided their written informed consent to participate in this study.

## Author contributions

EW-S, MZ, and AB designed the study. EW-S wrote the first draft of the manuscript. EW-S, AB, MZ, AM-B, KN-B, and AF-G collected data and performed literature searches. MZ performed the statistical analyzes. EW-S, MZ, and KJ-R performed a critical revision of the manuscript for intellectual content. All authors have read and agreed to the published version of the manuscript.

## Conflict of interest

The authors declare that the research was conducted in the absence of any commercial or financial relationships that could be construed as a potential conflict of interest.

## Publisher’s note

All claims expressed in this article are solely those of the authors and do not necessarily represent those of their affiliated organizations, or those of the publisher, the editors and the reviewers. Any product that may be evaluated in this article, or claim that may be made by its manufacturer, is not guaranteed or endorsed by the publisher.
