# Supplementary material for: Coronavirus disease 2019 vaccination uptake and hesitancy among Polish patients with inborn errors of immunity, autoinflammatory syndromes, and rheumatic diseases: A multicenter survey
Source: Front Immunol. 2022 Oct 6;13:1010899. doi: 10.3389/fimmu.2022.1010899 (PMC9582147; doi:10.3389/fimmu.2022.1010899)
Supplement: Supplementary file 1 [file Table_1.docx]

Study questionnaire "Attitude towards prophylactic vaccinations against SARS-CoV-2 virus in adult immunocompromised patients"

1. Date of completing the questionnaire

………………………….

2. Year of birth

…………………………

3. Gender

- Female
- Male

4. Type of immune disorder

- Common variable immunodeficiency (CVID)
- Agammaglobulinemia
- Selective IgA deficiency
- Deficiency of IgG subclasses
- Specific antibodies deficiency
- Unspecified hipogammaglobulinemia
- Autoinflammatory disease:
- Another disease: ………………………..

5. Education

- Primary
- Vocational
- Secondary
- Higher

6. Professional activity

- White-collar worker
- Green-collar worker
- Unemployed
- Pensioner
- Pupil / student
- Other

7. Place of residence

- City
- Village

8. Voivodeship

- Lower Silesia
- Kuyavian-Pomeranian
- Lublin Province
- Lubuskie Province
- Lodz Province
- Lesser Poland
- Masovian
- Opole Province
- Podkarpackie
- Podlasie
- Pomeranian
- Silesian
- Świętokrzyskie Province
- Warmia and Mazury
- Greater Poland
- West Pomeranian

9. Are you treated with immunoglobulins?

- Yes, intravenous treatment
- Yes, subcutaneous treatment
- No

10. Do you take immunoglobulins at home?

- Yes
- No.

11. Do you take any immunosuppressive treatment?

- Yes,
- No.
- I don't know

12. Do you receive biologic treatment?

- Yes, (please specify name of medication) …………………………………… ..
- No.
- I don't know

13. How many infections have you had in the last 3 months?

……………………………………….

14. Have you taken an antibiotic in the last 3 months?

- Not at all
- 1 time
- 2 or more times
- Constantly – infections prevention

15. Do you have any other chronic diseases?

- Yes
- No

16. Do you think that you are at risk of severe COVID 19 course?

- Yes
- No
- I don’t know

17. Did you suffer from COVID-19 disease?

- Yes, treatment in the hospital
- Yes, treatment at home
- No

18. Have your family members / relatives /housemates suffered from COVID-19?

- Yes, treatment in the hospital
- Yes, treatment at home
- No

19. Are you vaccinated against COVID-19?

- Yes, two doses
- Yes, one dose
- No

20. Vaccine manufacturer

- Pfizer
- Moderna
- Astra Zeneca
- Johnson & Johnson

21. Will you want to get another (booster) dose of the COVID-19 vaccine if it is possible and recommended?

- Yes
- No
- I don’t know

22. Have you got any adverse vaccination event?

- No.
- Yes, minor (swelling, redness, pain, fever, weakness)
- Yes, severe (shock, hospital or emergency room after vaccination)
- Other

23. What influenced your decision to vaccinate (multiple options can be chosen)?

- My own opinion that it is correct decision
- Fear of getting COVID-19
- Expert opinion
- Opinion of relatives and friends
- Other: ……………………………………………………………………………….

24. Do you think that vaccination against COVID-19 is safe?

- Yes
- Yes, but only for healthy people
- I don't know
- No.

25. In your opinion vaccination against COVID-19 is effective in persons with your disease?

- Yes
- No.
- I don't know

26. Are you afraid that vaccination against COVID-19 may flare/worsen your disease?

- Yes
- No
- I don't know

27. Should COVID-19 vaccination be mandatory? (you can choose multiple answers)?

- No.
- Yes, for everyone
- Yes, for selected professional groups
- Yes, for people at risk of severe disease course

28. Have you been vaccinated against influenza?

- Yes, every year
- One or more
- Never

29. Are you considering changing your COVID-19 vaccination decision in the future?

- Yes
- No

30. Are your closest family or housemates vaccinated?

- Yes
- Yes, but only because of me
- No
- I don't know

The Brief Illness Perception Questionnaire

For the following questions, please circle the number that best corresponds to your views:

1. **How much does your illness affect your life?**

0 1 2 3 4 5 6 7 8 9 10

no affect at all severely affects my life

1. **How long do you think your illness will continue?**

0 1 2 3 4 5 6 7 8 9 10

a very short time forever

1. **How much control do you feel you have over your illness?**

0 1 2 3 4 5 6 7 8 9 10

Absolutely no control extreme amountof control

1. **How much do you think your treatment can help your illness?**

0 1 2 3 4 5 6 7 8 9 10

not at all extremely helpful

1. **How much do you experience symptoms from your illness?**

0 1 2 3 4 5 6 7 8 9 10

no symptoms at all many severe symptoms

1. **How concerned are you about your illness?**

0 1 2 3 4 5 6 7 8 9 10

not at all concerned extremely concerned

1. **How well do you feel you understand your illness?**

0 1 2 3 4 5 6 7 8 9 10

don’t understand at all understand very clearly

1. **How much does your illness affect you emotionally? (e.g. does it make you angry, scared, upset or depressed?)**

0 1 2 3 4 5 6 7 8 9 10

not at all affected emotionally extremely affected emotionally

**Please list in rank-order the three most important factors that you believe caused your illness.**

**The most important causes for me:**

1. .................................................................

2. ...............................................................

3. ...............................................................
